# On the Performance of Video Resolution, Motion and Dynamism in Transmission Using Near-Capacity Transceiver for Wireless Communication

**DOI:** 10.3390/e23050562

**Published:** 2021-05-01

**Authors:** Nasru Minallah, Khadem Ullah, Jaroslav Frnda, Laiq Hasan, Jan Nedoma

**Affiliations:** 1 Department of Computer Systems Engineering, University of Engineering and Technology Peshawar, Peshawar 25000, Pakistan; n.minallah@uetpeshawar.edu.pk (N.M.); 14pwcse1224@uetpeshawar.edu.pk (K.U.); laiqhasan@uetpeshawar.edu.pk (L.H.); 2 Department of Quantitative Methods and Economic Informatics, Faculty of Operation and Economics of Transport and Communications, University of Zilina, 010 26 Zilina, Slovakia; jaroslav.frnda@fpedas.uniza.sk; 3 Department of Telecommunications, Faculty of Electrical Engineering and Computer Science, VSB—Technical University of Ostrava, 17. Listopadu 15, 708 33 Ostrava-Poruba, Czech Republic

**Keywords:** H.265 High Efficient Video Coding (HEVC), Sphere Packing (SP) Modulation, BER reduction, Differential Space Time Spreading (DSTS), Extrinsic Information Transfer (EXIT) Chart

## Abstract

This article investigates the performance of various sophisticated channel coding and transmission schemes for achieving reliable transmission of a highly compressed video stream. Novel error protection schemes including Non-Convergent Coding (NCC) scheme, Non-Convergent Coding assisted with Differential Space Time Spreading (DSTS) and Sphere Packing (SP) modulation (NCDSTS-SP) scheme and Convergent Coding assisted with DSTS and SP modulation (CDSTS-SP) are analyzed using Bit Error Ratio (BER) and Peak Signal to Noise Ratio (PSNR) performance metrics. Furthermore, error reduction is achieved using sophisticated transceiver comprising SP modulation technique assisted by Differential Space Time Spreading. The performance of the iterative Soft Bit Source Decoding (SBSD) in combination with channel codes is analyzed using various error protection setups by allocating consistent overall bit-rate budget. Additionally, the iterative behavior of SBSD assisted RSC decoder is analyzed with the aid of Extrinsic Information Transfer (EXIT) Chart in order to analyze the achievable turbo cliff of the iterative decoding process. The subjective and objective video quality performance of the proposed error protection schemes is analyzed while employing H.264 advanced video coding and H.265 high efficient video coding standards, while utilizing diverse video sequences having different resolution, motion and dynamism. It was observed that in the presence of noisy channel the low resolution videos outperforms its high resolution counterparts. Furthermore, it was observed that the performance of video sequence with low motion contents and dynamism outperforms relative to video sequence with high motion contents and dynamism. More specifically, it is observed that while utilizing H.265 video coding standard, the Non-Convergent Coding assisted with DSTS and SP modulation scheme with enhanced transmission mechanism results in $E_{b}/N_{0}$ gain of 20 dB with reference to the Non-Convergent Coding and transmission mechanism at the objective PSNR value of 42 dB. It is important to mention that both the schemes have employed identical code rate. Furthermore, the Convergent Coding assisted with DSTS and SP modulation mechanism achieved superior performance with reference to the equivalent rate Non-Convergent Coding assisted with DSTS and SP modulation counterpart mechanism, with a performance gain of 16 dB at the objective PSNR grade of 42 dB. Moreover, it is observed that the maximum achievable PSNR gain through H.265 video coding standard is 45 dB, with a PSNR gain of 3 dB with reference to the identical code rate H.264 coding scheme.

## 1. Introduction

Video on-demand transmission is the major factor of the estimated increase in network traffic over the cellular network. By the end of 2023, the emerging increase in mobile applications downloading will reach 300 million and the 5G speeds will reach about 575 Mbps, which is 13 times higher than the average mobile connections. There will be a significant need in increase of bandwidth with the highest connectivity requirements of the future networks. A crucial factor leading the increase in mobile speed is the increasing proportion of 4G mobile connection and the rising interest in 5G connections [1,2,3]. The applications of Internet data traffic on 3G and 4G over wireless communication networks increases exponentially, which increases the needs of highly efficient networks providing superior connectivity, reliable and low latency communication [4].

For a raw video sequence with one pixel representation of a gray scale and color video, 8 and 24 bits are used correspondingly. A total of 944 Mbps is required to display one frame of a simple colored TV video with a resolution of 1024 × 768 pixels with a frame rate of 25 fps [5]. Bandwidth requirement is reduced by encoding the video content through source video coding standards such as H.264/AVC or H.265/HEVC for transmission over the communication network. However, the High Definition (HD) videos require higher bandwidth for transmission over communication channels, even with the employment of the video compression mechanism.

For the very first time, Shannon proposed the basics of channel capacity bounds over a noisy channel providing minimum probability of errors by employing complex encoding systems and identified the error-resilient features of the communication systems [6,7]. Shannon’s coding theorem presented signals and messages as a point in space and developed a method that any communication system can be represented geometrically. After that researcher presented different techniques for achieving that limit. The first forward error correction block code for single error correction was the Hamming code [8]. Elias was the first who discovered error-correcting convolutional codes [9,10], in which the encoding dependencies are shifted from finite-length segments to encoding dependencies that exist over the entire block. The authors in [11] used convolutional codes for the burst of error correction in information bit-stream. In [12], sequential algorithms were proposed for decoding the convolutional codes. A heuristic discussion was presented in greater detail about probabilistic decoding [13]. Furthermore, the authors presented in depth analysis about A-priori, A-posteriori knowledge, mutual information, channel degradation, channel quantization and channel capacity i.e., different terminologies linked with designing an encoding and decoding schemes. The Viterbi Algorithm (VA) proposed in [14] is a significant achievement ever in the history of convolutional correction codes. VA primarily works on finding the closest sequence of the transmitted information bits (Maximum A-posteriori Probability (MAP) sequence estimation), which results in minimum Bit Error Ratio (BER) [15,16]. VA can also be used as a maximum likelihood sequence detector for AWGN channel, which finds applications in Code Division Multiple Access (CDMA), Time Division Multiple Access (TDMA), and various other fields [16,17]. An efficient bidirectional search algorithm for computing the free distance of a convolutional codes is described in [18]. VA works on codeword error minimization for convolutional codes. Therefore, authors proposed the symbol based MAP algorithm in [19], minimizing error rate in symbols or bits through optimal decoding. However, compared to VA, the symbol based MAP decoding is only attractive for short block codes with short constraint lengths due to offering higher complexity algorithm and large storage for larger constraint lengths. Turbo codes are first presented in [20,21], which comprises the concatenation of two Recursive Systematic Convoluational (RSC) codes interfaced through an interleaver. Turbo codes are also used in third-generation (3G) mobile radio systems [22]. In turbo codes, an iterative algorithm is used at the decoder side to extract the transmitted information bits. The authors proposed Soft Output Viterbi Algorithm (SOVA) in [23]. They modified the VA in such a way that it gives the most probable transmitted sequence in a Markov finite-state chain along with the reliability or posteriori information. Koch et.al in [12] proposed the Max Log MAP algorithm for turbo decoder with lower complexity than SOVA.

Authors in [24,25,26,27,28] evaluated the performance of the Joint Source Channel Codes (JSCC) and it was concluded that it could be jointly optimized as a one pair for achieving a lower BER. Authors in [24] proposed an extended curve-fitting algorithm which finds optimal design criteria for JSCC. In [29], the authors used and extended the application of Fully Parallel Turbo Decoder (FPTD), for the implementation of a unary error correction on hardware considering the video transmission of information bits on JSCC. An analog low complexity JSCC system is designed [30] for the transmission of still images. Deep JSCC is proposed [31], which does not explicitly relay on source and channel encoder and instead trained an auto-encoder composed of two convolutional neural network and can be used in a non-trainable layer in the middle of a communication channel. In [32], the authors proposed a lower complexity Robust Distributed Video Coding (RDVC) framework to optimize the quality of video communication for wireless multimedia sensors networks. A new coding scheme is presented based on Wyner-Ziv coding for error resilience and Rate Distortion (RD) performance. In [33], the authors proposed systematic code Low Density Generator Matrix (LDGM), which under maximum likelihood decoding, can achieve the capacity of memoryless binary input/output channel. The extent of performance achievement in iterative decoding is determined from the number of profitable iterations by EXIT chart analysis in [34]. In [35], the authors showed the EXIT chart as a versatile tool for designing of different serial concatenation codes. The authors [36] proposed multi-edge type EXIT chart bit mapping for low density parity check Bit-interleaved Coded Modulation (BiCM).

A delightful source coding candidate for wireless video communication is the compression efficient standard H.264 [37,38,39,40]. In [41], the authors proposed a mobile model called Proposed Generation (Pro-G) for supporting large number of user applications, making usage of wider bandwidth, adoptive modulation and coding for cellular systems. Furthermore, transcoding technique (H.265 pro, incorporating two Highest Efficiency Video Coding (HEVC) structure) is used for adoptive video streaming, which results in providing multiple data rates of a video streams. Authors in [42] studied the compressed video transmission using JSCC based on the stream contents characteristics of HEVC. Clustering algorithm Fuzzy C-Means (FCM) from the field of artificial intelligence is used for reducing the noise effects and for classifications of wireless channel. A new Sample Adaptive Offset (SAO) in loop filter make feasible the higher compression efficiency of H.265/HEVC for both objective and subjective measures. Estimating the best SAO parameter per coding tree unit for low powered or real time encoders results in a number of issues such a high computational complexity or architectural inefficiency. The authors proposed in [43] various SAO policies reducing complexity and removing the inefficiency caused by its implementation. A broadcaster group from Japan plan to stream a television services based on the latest new standard HEVC having twice the compression capability compared to H.264. A studied has been carried out for estimating the required bit rates. Different multiformat videos were evaluated in [44] and suggested that a 10–15 Mbits/s is required for 1080/60/I and 1080/60/P, 30–40 Mbit/s is required for 2160/60/P, while 80–100 Mbit/s is needed for 4320/60/P format. The study deduce a conclusion that such videos can also be transmitted via the existing satellite channel bandwidth. The authors in [45] proposed a method for identifying the moving objects that passes through the specific region without fully decoding the bitstreams. Foreground prediction block is extracted from the video bit-stream according to the motion vector of H.265 and clustered into region of interests. The state of moving object can be find by match the moving objects and the region of interest in the current frame. HEVC chips are incorporated into the application processor system-on-a-chip within the mobile devices due to its wide usage in video transmission. However, the coding bandwidth accessed for the motion estimation (ME) operation within HEVC has changed over time due to the adaption of the intelligent power management with in the process system-on-a-chip [46]. The needs of on-demand coding bandwidth for HEVC must be considered in designing a low power system. The authors proposed an intelligent ME controller algorithm model, which is coding bandwidth efficient and referred to a Dynamic Voltage Frequency Scale aware (DVFS-aware). DVFS-aware can be integrated in ME for coding bandwidth realization, coding bit-rate, and coding-quality-optimized HEVC ME design. The authors in [47] proposed an algorithm for improving the bitrate (<; 1.5 %), while a slight decrease in PSNR (<; 0.15 decibel) has been reported.

Taking in mind the above background, we propose an arrangement for the H.264 and HEVC compressed video bitstream transmission through Soft Bit Source Decoding (SBSD) scheme. The propose system uses H.264 and H.265 [48] as a source encoder for simulation purpose. Artificial redundancy is generated in the transmitter side using two different combination of Over Complete Mapping (OSM) and RSC encoder while keeping the overall bitrate constant. The redundancy is iteratively utilized in the decoder side for the enhancement of BER performance. Its hard to estimate an impulse response for each individual Multiple Input Multiple Output (MIMO) link in Rayleigh fading channel due to experiencing different fading. In order to further improve the performance of the system, a transmitter diversity gain technique is incorporated, such that the coded video bitstream is passed to Sphere Packing (SP) modulation and Differential Space Time Spreading (DSTS) scheme which overcome channel estimation dependency. Due to embedding DSTS MIMO scheme, the proposed transceivers can be deployed in diverse environments for providing better performance.

The following is the main contributions of the proposed work while considered in designing of the three systems i.e., NCC, NCDSTS-SP and CDSTS-SP:

- H.264 and H.265 source compression standard has been incorporated in order to visualize the quality of the highly compressed video stream at the receiver.
- SP modulation are included in order to observe the performance of the proposed systems on fading channel.
- SP modulation is inspired by space time modulation and provides the diversity and coding gain for the proposed system in order to efficiently estimate and recover the actual transmitted information.
- DSTS scheme is included to remove the Channel State Information (CSI) estimation dependency and the receiver does not required to know the channel fade.
- Different inner and outer code rates are used in the concerned schemes to visualize the effect of code rates on the convergence property of iterative schemes.
- EXIT chart are used for finding the number of profitable iterations in the decoding process.
- Diverse video sequences have been tested to measure the role of the static objects having fine details, dynamism and motion of the object in the background, on the performance metrics.
- To the best of our knowledge, this is distinctive research study to transmit the highest compression efficient coded video using the H.265/HEVC standard while employing the advocated wireless transmission setup.

The rest of the proposed manuscript has been structured as follows. In Section 2, preliminaries and system design criteria has been presented that is used in designing of our proposed systems. Section 3, a detailed analysis of the proposed system models has been briefly explained. Section 4 presents the performance analysis of our propose simulation. Finally, a conclusion of the resultant work is provided in Section 5.

## 2. Preliminaries & System Design Criteria

MIMO schemes are mainly used for multiplexing and diversity gain. In multiplexing, different uncorrelated symbols are transmitted from all the antennas while for providing diversity gain, the transmitted symbols should be correlated. Space Time Block Codes (STBC) performs encoding both in space domain as well as in time domain due to using two transmitting (Tx) antennas and requiring two period for transmitting the information symbols. The first Tx antenna transmit the original symbol and all the remaining Tx antennas are transmitting the linear combination or the conjugate version of the symbol that are transmitting from the first antennas. In case of two Tx antennas, symbols $d_{1}$ and $d_{2}$ are transmitted at the first time period $t_{1}$ while at time period $t_{2}$, $-d_{2}^{*}$ and $d_{1}^{*}$ are transmitted, for which the decoded matrix can be represented as [49]:

$$\left[\begin{array}{cc} d_{1} & -d_{2}^{*}\\ d_{2} & d_{1}^{*} \end{array}\right]$$

From the decoding matrix, the column contains the symbols transmitted at the corresponding time period while the rows comprises of the symbols transmitted from the specific Tx antenna. STBC with the following encoding matrix was proposed for four Tx antennas [50].

(1) $$\frac{1}{\sqrt{2}}\cdot [\begin{array}{ccccc} d_{1} & -d_{2}^{*} & -z_{1}^{*} & -z_{2}^{*} & \\ d_{2} & d_{1}^{*} & z_{2} & -z_{1}\\ d_{3} & -d_{4}^{*} & d_{1}^{*} & d_{2}^{*}\\ d_{4} & d_{3}^{*} & -d_{2} & d_{1} \end{array}]$$

where $d_{1}$, $d_{2}$, $d_{3}$, and $d_{4}$ are the data symbols, $z_{1}=Real\left(d_{3}\right)-j\;imaginary\left(2d_{1}d_{2}d_{4}^{*}\right)$ and $z_{2}=d_{1}^{*}+d_{4}+d_{2}^{2}d_{4}^{*}+d_{1}^{*}d_{2}x_{3}-d_{1}^{*}d_{2}d_{3}*$. Differential Space Time Block Codes (DSTBC) does not required CSI estimation at both the Tx or receiving (Rx) antenna. In fast fading channel, the training sequences becomes outdated. Therefore, the overhead will increases if we are transmitting the training sequence at each instance. Differential scheme is suitable in case of fading channels. For PSK modulation having $M_{sp}$ signal points and spectral efficiency $m=\log_{2}M_{sp}$, the symbols set $Sym_{t}$ for a transmission period of *t* using differential PSK from the constellation are:

(2) $$Sym_{t}=\exp\left(j\frac{2\pi \psi_{t}}{M_{sp}}\right)$$

where $\psi_{t}\epsilon \left\{0,1,2,3\ldots M_{sp}-1\right\}$. Therefore, the differential encoder, encodes the symbol in such a way that the data information is the difference between the phases of the current and previous symbols such that $d_{t}=Sym_{t}\cdot d_{t-1}$. Similarly, DSTBC following the same method for two Tx antennas in transmitting the two symbols having phases $d_{t+1}$, $d_{t+2}$ correspondingly, and the previous transmitted symbols $d_{t-1}$, $d_{t}$. Therefore, in each block, the sum of the phases of new vector ${\left[\begin{array}{cc} d_{t+1} & d_{t+2} \end{array}\right]}^{T}$ and the previously transmitted vectors, ${\left[\begin{array}{cc} d_{t-1} & d_{t} \end{array}\right]}^{T}$ and ${\left[\begin{array}{cc} -d_{t}^{*} & d_{t-1}^{*} \end{array}\right]}^{T}$, is sent as follow [49]:

(3) $$\left[\begin{array}{c} d_{t+1}\\ d_{t+2} \end{array}]=[\begin{array}{cc} d_{t-1}^{*} & d_{t}^{*}\\ -d_{t} & d_{t-1} \end{array}]\cdot [\begin{array}{c} Sym_{t+1}\\ Sym_{t+2} \end{array}\right]$$

The design criteria that guarantee maximum diversity and coding gain can be find [49], e.g., the signal received (${r_{sig}}_{t}^{j}$) at antenna j after demodulation is expressed as in the following equation [51].

(4) $${r_{sign}}_{t}^{j}=\sum_{i=1}^{N}\alpha_{i,j}\psi_{t}^{i}\sqrt{E}+\eta_{t}^{j}$$

Here, the codeword is expressed with $\psi_{t}^{j}$ and the path gain is represented with $\eta_{t}^{j}$. The relationship in Equation (4) between input and output can be also referred to fading channel model. The codewords that are transmitting from a total of *N* Tx antennas over the total duration of *T* time period transmission is given below.

(5) $$\psi^{1}=\left(\begin{array}{cccc} \psi_{1,1}^{1} & \psi_{1,2}^{1} & \cdots & \psi_{1,N}^{1}\\ \psi_{2,1}^{1} & \psi_{2,2}^{1} & \cdots & \psi_{2,N}^{1}\\ \vdots & \vdots & \ddots & \vdots \\ \psi_{T,1}^{1} & \psi_{T,2}^{1} & \cdots & \psi_{T,N}^{1} \end{array}\right)$$

At the receiving side, the signal for a *T* time period of transmission with a total of *M* receiving antennas is expressed as in the following equation.

(6) $${r_{sig}}_{t}=\left(\begin{array}{cccc} {r_{sig}}_{1,1} & {r_{sig}}_{1,2} & \cdots & {r_{sig}}_{1,M}\\ {r_{sig}}_{2,1} & {r_{sig}}_{2,2} & \cdots & {r_{sig}}_{2,M}\\ \vdots & \vdots & \ddots & \vdots \\ {r_{sig}}_{T,1} & {r_{sig}}_{T,2} & \cdots & {r_{sig}}_{T,M} \end{array}\right)$$

If the antennas are far away then the path gain is independent from each other, otherwise a spatial correlation exist that can be expressed with the following N×M channel matrix:

(7) $$H=\left(\begin{array}{cccc} \alpha_{1,1} & \alpha_{1,2} & \cdots & \alpha_{1,M}\\ \alpha_{2,1} & \alpha_{2,2} & \cdots & \alpha_{2,M}\\ \vdots & \vdots & \ddots & \vdots \\ \alpha_{N,1} & \alpha_{N,2} & \cdots & \alpha_{N,M} \end{array}\right)$$

(8) r=ψ·H+N

where noise N of a T×N matrix is expressed as given below.

(9) $$N=\left(\begin{array}{cccc} \eta_{1,1} & \eta_{1,2} & \cdots & \eta_{1,M}\\ \eta_{2,1} & \eta_{2,2} & \cdots & \eta_{2,M}\\ \vdots & \vdots & \ddots & \vdots \\ \eta_{T,1} & \eta_{T,2} & \cdots & \eta_{T,M} \end{array}\right)$$

An error occurred if codeword $\psi_{1}$ is selected from the codebook at the transmitter antenna and the receiver estimates $\psi_{2}$ due to some noise present in the channel.

(10) $$\psi^{2}=\left(\begin{array}{cccc} \psi_{1,1}^{2} & \psi_{1,2}^{2} & \cdots & \psi_{1,N}^{2}\\ \psi_{2,1}^{2} & \psi_{2,2}^{2} & \cdots & \psi_{2,N}^{2}\\ \vdots & \vdots & \ddots & \vdots \\ \psi_{T,1}^{2} & \psi_{T,2}^{2} & \cdots & \psi_{T,N}^{2} \end{array}\right)$$

The probability of error using the union bound for a set of comprising *L* number of codewords is given below:

(11) $$P\left(\;\mathrm{error}\;|\psi^{1}\;\mathrm{is}\;\mathrm{sent}\;\right)\leq \sum_{i=2}^{L}P\left(\psi^{1}\to \psi^{i}\right)$$

For an error matrix $D\left(\psi_{1},\psi_{2}\right)=\psi_{2}-\psi_{1}$, the pairwise error probability to be define [51] in term of non negatives reals number eigenvalues $\lambda_{n}\geq 0$ of matrix $A\left(\psi_{1},\psi_{2}\right)=D{\left(\psi_{1},\psi_{2}\right)}_{H}\cdot D\left(\psi_{1},\psi_{2}\right)$ $={\left(\psi_{2}-\psi_{1}\right)}_{H}\cdot \left(\psi_{2}-\psi_{1}\right)$ where *H* is conjugate and transpose a the corresponding matrix.

(12) $$P\left(\psi \to \mathrm{error}\right)\leq {\left(\prod_{n=1}^{r}\lambda_{n}\right)}^{-M}{\frac{E_{s}}{4N_{0}}}^{-rM}$$

or

(13) $$P\left(\psi^{1}\to \psi^{2}\right)\leq \frac{{\left(\frac{E_{s}}{4N_{0}}\right)}^{-rM}}{{\left(\prod_{n=1}^{r}\lambda_{n}\right)}^{M}}$$

or

(14) $$P\left(\psi^{1}\to \psi^{2}\right)\leq \frac{4^{rM}}{{\left(\prod_{n=1}^{r}\lambda_{n}\right)}^{M}\gamma^{rM}}$$

The rank of the matrix $A(\psi_{1},\psi_{2})$ represent the diversity gain of the space time codes which can be also expressed with the rank of the difference matrix $D(\psi_{1},\psi_{2})$ multiplied with the number of *M* receiving antennas, i.e., the power of SNR (r×M) in the denominator of Equation (14). A rank cafeteria to be defined for guarantee a full diversity scheme as, if the matrix $A(\psi_{i},\psi_{i})$ is a full rank matrix for all the possible codewords. Contrary to diversity gain, coding gain is the distance between two codes which is related to the determinate of matrix A(ψ1,ψ2). Coding gain can be improve by maximizing the minimum determinate of matrix A(ψ1,ψ2) [49,51].

The Alamouti code is a full rank matrix and is satisfies the determinant criteria. Considering a time correlated Rayleigh fading channel, SP modulation obtaining a full diversity gain using the joint combination of orthogonal designs with sphere packing [52]. Let, the orthogonal function $G1\left(d\right)=d_{1}I_{1}$, where $I_{1}$ denotes the identity matrix, then the recursive orthogonal function is expressed as given below [52,53]:

(15) $$G_{2^{k}}\left(d_{1},\cdots ,d_{k+1}\right)=\left[\begin{array}{cc} G_{2^{k-1}}\left(d_{1},\cdots ,d_{k}\right) & d_{k+1}I_{2^{k-1}}\\ -d_{k+1}^{*}I_{2^{k-1}} & G_{2^{k-1}}^{H}\left(d_{1},\cdots ,d_{k}\right) \end{array}\right]$$

where $d_{1},d_{2},\ldots d_{k+1}$ are the complex variables and their conjugate is expressed as $d_{1}^{*},d_{2}^{*},\ldots d_{k+1}^{*}$, H is the transpose and conjugate of $G_{2^{k-1}}$.

Furthermore, the SP signals are constructed using the above equation as expressed in the following equation.

(16) $$Sp=\sqrt{2^{k}/\left(k+1\right)}G_{2^{k}}\left(d_{1},d_{2},\cdots ,d_{k+1}\right)$$

The symbol rate is denote as $k+1/2^{k}$ where the $\sqrt{2^{k}/\left(k+1\right)}$ is used for energy constraint as a normalization factor. Using Equation (16), the set of SP signals for 2 transmitting antennas are given below:

(17) $$Sp=\sqrt{2^{k}/\left(k+1\right)}G_{2}\left(d_{1},d_{2}\right)=$$

$$\sqrt{2^{k}/\left(k+1\right)}\left[\begin{array}{cc} G_{1}\left(d_{1}\right) & d_{2}\\ -d_{2}^{*} & G_{1}^{H}\left(d_{1}\right) \end{array}\right]=\sqrt{2^{k}/\left(k+1\right)}\left[\begin{array}{cc} d_{1} & d_{2}\\ -d_{2}^{*} & d_{1}^{*} \end{array}\right]$$

Using the same Equation (16), the set of SP signals can be constructed for four and eight transmitting antennas is given below:

(18) $$Sp=\sqrt{2^{k}/\left(k+1\right)}G_{4}\left(d_{1},d_{2},d_{3}\right)=$$

$$\sqrt{2^{k}/\left(k+1\right)}\left[\begin{array}{cc} G_{2}\left(d_{1},d_{2}\right) & d_{3}I_{2}\\ -d_{3}^{*}I_{2} & G_{2}^{H}\left(d_{1},d_{2}\right) \end{array}\right]$$

$$=\sqrt{2^{k}/\left(k+1\right)}\left[\begin{array}{cccc} d_{1} & d_{2} & d_{3} & 0\\ -d_{2}^{*} & d_{1}^{*} & 0 & d_{3}\\ -d_{3}^{*} & 0 & d_{1}^{*} & -d_{2}\\ 0 & -d_{3}^{*} & d_{2}^{*} & d_{1} \end{array}\right]$$

(19) $$Sp=\sqrt{2^{k}/\left(k+1\right)}G_{8}\left(d_{1},d_{2},d_{3},d_{4}\right)=$$

$$\sqrt{2^{k}/\left(k+1\right)}\left[\begin{array}{cc} G_{4}\left(d_{1},d_{2},d_{3}\right) & d_{4}I_{4}\\ -d_{4}^{*}I_{4} & G_{4}^{H}\left(d_{1},d_{2},d_{3}\right) \end{array}\right]$$

$$=\sqrt{2^{k}/\left(k+1\right)}\left[\begin{array}{cccccccc} d_{1} & d_{2} & d_{3} & 0 & d_{4} & 0 & 0 & 0\\ -d_{2}^{*} & d_{1}^{*} & 0 & d_{3} & 0 & d_{4} & 0 & 0\\ -d_{3}^{*} & 0 & d_{1}^{*} & -d_{2} & 0 & 0 & d_{4} & 0\\ 0 & -d_{3}^{*} & d_{2}^{*} & d_{1} & 0 & 0 & 0 & d_{4}\\ -d_{4}^{*} & 0 & 0 & 0 & d_{1}^{*} & -d_{2} & -d_{3} & 0\\ 0 & -d_{4}^{*} & 0 & 0 & d_{2}^{*} & d_{1} & 0 & -d_{3}\\ 0 & 0 & -d_{4}^{*} & 0 & d_{3}^{*} & 0 & d_{1} & d_{2}\\ 0 & 0 & 0 & -d_{4}^{*} & 0 & d_{3}^{*} & -d_{2}^{*} & d_{1}^{*} \end{array}\right].$$

**Figure 1:**
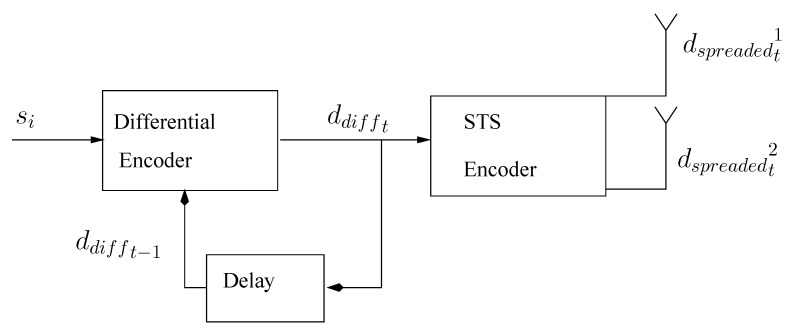
Differential Space Time Spreading Scheme.

The differential MIMO (DSTS) scheme comprises two encoders i.e., differential encoder and space time spreading (STS) encoder as shown in Figure 1. The symbols are first differential encoded and then spreaded by STS scheme. For the very first time, two dummy symbols ${d_{\mathrm{diff}}}_{0}^{1}$, ${d_{\mathrm{diff}}}_{0}^{2}$ are transmitted which have no information bits. After that, the data information stream $d_{t}^{k};k=1,2$ are separated into ${d_{\mathrm{diff}}}_{t}^{1}$, ${d_{\mathrm{diff}}}_{t}^{2}$ symbols. The encoded symbols at the 2 transmitter antennas are then expressed as with the following equation [54].

(20) $${d_{\mathrm{diff}}}_{t}^{1}=\frac{d_{t}^{1}\cdot {d_{\mathrm{diff}}}_{t-1}^{1}+x_{t}^{2}\cdot {d_{\mathrm{diff}}}_{t-1}^{2*}}{\sqrt{{|{d_{\mathrm{diff}}}_{t-1}^{1}|}^{2}+{|{d_{\mathrm{diff}}}_{t-1}^{2}|}^{2}}}$$

(21) $${d_{\mathrm{diff}}}_{t}^{2}=\frac{d_{t}^{1}\cdot {d_{\mathrm{diff}}}_{t-1}^{2}-d_{t}^{2}\cdot {d_{\mathrm{diff}}}_{t-1}^{2*}}{\sqrt{{|{d_{\mathrm{diff}}}_{t-1}^{1}|}^{2}+{|{d_{\mathrm{diff}}}_{t-1}^{2}|}^{2}}}$$

**Figure 2:**
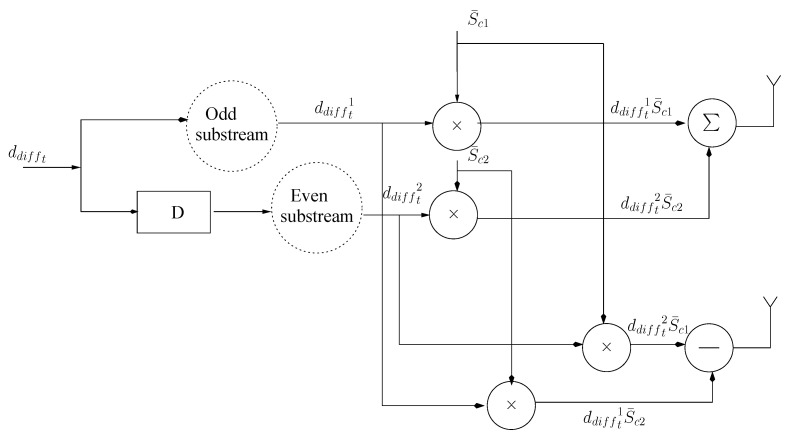
Space Time Spreading Technique.

After passing from the STS encoder, the two consecutive symbols at both the transmitting antennas is expressed with the following mapping as shown in Figure 2 [54].

(22) $${d_{spreaded}}_{t}^{1}=\frac{1}{\sqrt{2}}\left({\bar{S_{c}}}_{1}\cdot {d_{\mathrm{diff}}}_{t}^{1}+{\bar{S_{c}}}_{2}\cdot {d_{\mathrm{diff}}}_{t}^{2}\right)$$

(23) $${d_{spreaded}}_{t}^{2}=\frac{1}{\sqrt{2}}\left({\bar{S_{c}}}_{1}\cdot {d_{\mathrm{diff}}}_{t}^{2}-{\bar{S_{c}}}_{1}\cdot {d_{\mathrm{diff}}}_{t}^{2}\right)$$

where $\bar{{S_{c}}_{1}}$ and $\bar{{S_{c}}_{2}}$ are the orthogonal codes at both the transmitting antennas. The proposed work uses DSTS MIMO scheme for attaining diversity and coding gain. In case of Rayleigh fading fading channel, DSTS MIMO scheme is a suitable candidate for removing the CSI estimation at each instance of transmission.

## 3. Proposed System Model

**Figure 3:**
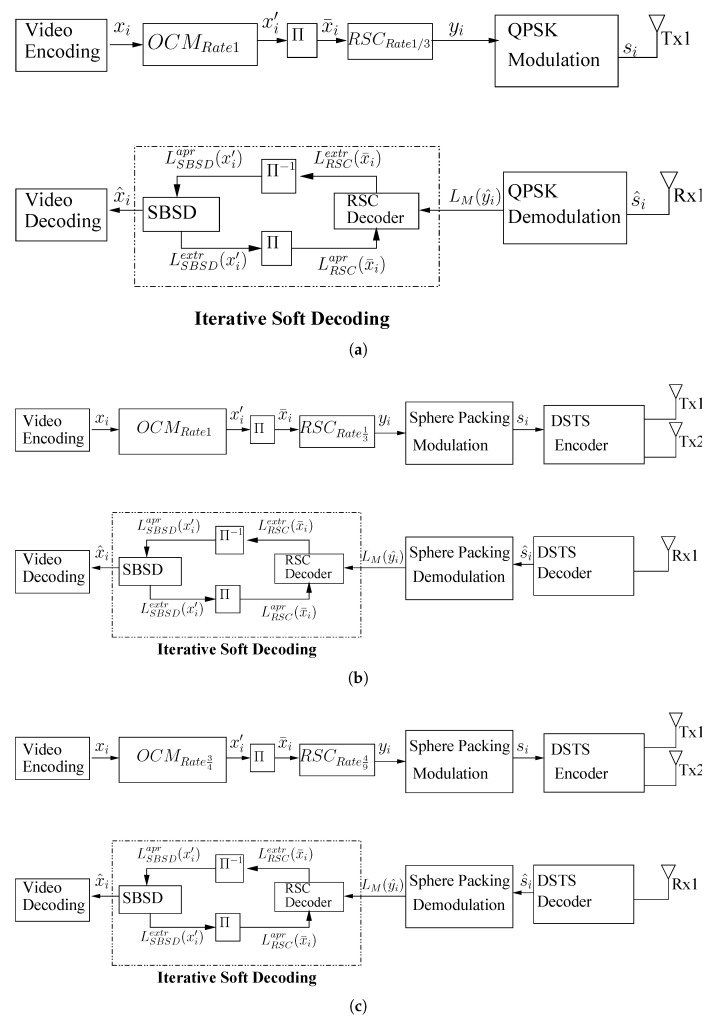
Proposed Systems (**a**) NCC; (**b**) NCDSTS-SP; (**c**) CDSTS-SP.

Typically, the primitive of the transmitter block comprises discrete input, encoder, digital modulator, and the output. Channel encoder is operating for the removal of error effects by adding some redundancy through a controlled methodology in the information bits. Channel flexibility takes place by manipulating RSC codes as a constituent channel codes to efficiently operate in AWGN and as well in Rayleigh fading channels. The preliminaries and system design criteria has been briefly explained in [26]. The proposed system model comprises three video phone arrangements as shown in Figure 3 and the specifications of the transceivers are given in Table 1, Table 2, Table 3, Table 4 and Table 5. All the transceivers follow the same mechanism and are only different by using different modulation schemes and arrangements of inner and outer code rates. In the proposed scheme, video is initially compressed at the transmitter side by using the international compression standard H.264 and H.265 video codec with the parameters settings of the source encoder as given in Table 1. The different building blocks of the proposed system models are as follows.

### 3.1. H.264 and H.265 Source Video Coding Standard

Advance Video Coding (AVC)/H.264 is a compression standard resulted in the extension of the video service delivery to various networks and applications. The coding architecture of H.264 consists of two basics layers i.e., Video Coding Layer (VCL) and Network Adaptation Layer (NAL). VCL provided efficient representation of video contents while NAL provides network friendly representation. On the other hand, HEVC is the latest state of the art video compression standard designed for achieving the goals of parallel-processing, resilience to data-loss and coding efficiency [55]. It comprises of a high-level syntax for supporting network interface, implementation aspects and a compressed data of video layer. HEVC has been extended with enhanced bit depth capability, scalability of embedded-bits and 3D multi-view representations [56]. The need of H.265 was realized due to the increasing demand of HD videos, higher resolutions videos and for the transmission of multi-view or 3D displays, which encouraged the joint collaborative team to bring new compression standard superior to prior codecs and to incorporate higher usage of parallel processing [57]. The primary goal in designing of HEVC was to reduce the number of bits for the same quality of video, compared to the predecessor standards. Some features retained in the H.265 standard architecture from H.264 standard are:

- Parameter set structure, containing sharable information deployed in the decoding process. The new Video Parameter Set (VPS) is based on the concepts of sequence and picture parameter sets used in H.264.
- Network Abstraction Layer (NAL) unit is retained which is helpful in identification of the purpose of payload data.
- In case of data losses, synchronization is done in H.265 with the help of slices. The same concept of slices is followed in H.264.
- Information about the timing of a video picture, interpretation of the color space and other display related information is provided by Supplemental Enhancement Information (SEI) and Video usability Information (VUI) metadata, adopted in H.265 and H.264.

The hybrid approach of standard H.261 concerning inter/intra-prediction and 2D transform is also imported in H.265. A prediction residual is computed based on the inter-picture motion compensation or intra-picture spatial predictions. This residual undergoes through block transformation, quantization and entropy coding respectively. A detailed description can be found in [55,56,58].

HEVC fulfilled the target 50% compression efficiency improvement over the predecessor H.264/AVC, providing adequate image quality with the trade-off computational complexity. HEVC consumes half bandwidth to that of the AVC thats why users experiences a reasonable video quality over very small bandwidth. The standard is specifically designed to be compatible with all the existing applications of the previous codecs.

**Figure 4:**
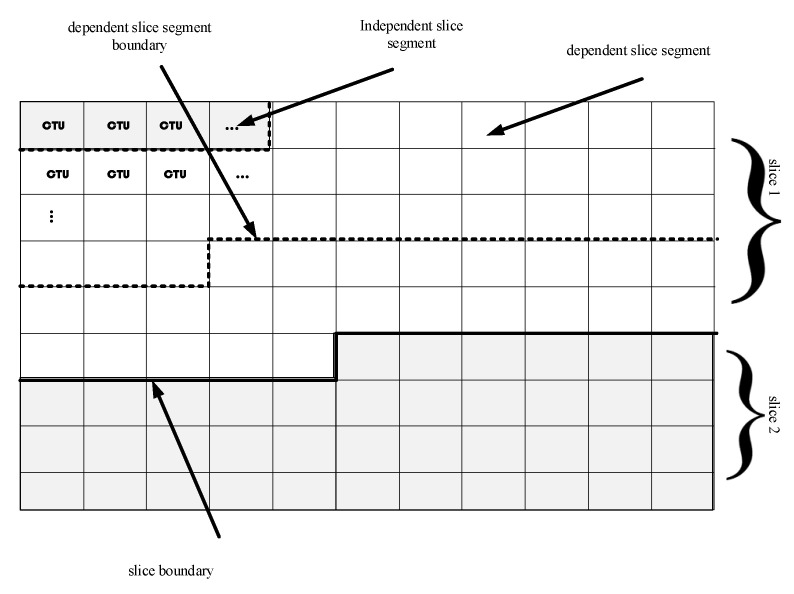
HEVC division of frame into slices.

Furthermore, HEVC divides each frame into slices where slice is a sequence of an independent and subsequent dependent slice segments. Each slice has a specific header that enables its independent decoding of all the other slices in a given picture. Slice segments comprises of Coding Tree Units (CTU) which contains one luma and two chroma Code Tree Block (CTB) along with their syntax while CTB is a region of N × N (N = 16, 32, 64) samples of the image, with the fact that larger sizes provide better compression. The higher size of the CTB is largely required when higher resolution videos are to be encoding which is beneficial for reducing the decoding time. Similarly, CTB can be further divided into coding blocks (CB), one luma CB and two Chroma CB along with syntax are called coding unit (CU). The size of CB should be in the range of 8 × 8 ≤ CB Size ≤ CTB size. The CTB blocks are then further divided into Transform Blocks (TB) for Discrete Cosine Transform (DCT) into a block of 4 × 4 and similarly the luma TBs and two chroma TBs combine along with their syntax to form Transform Unit (TU). For example, HEVC divides the frame into two slices while the first slice comprises three slice segments refer to Figure 4. The first slice are divided into 4 CTU independent segments, 32 depended segments and again 24 dependent segments, the second slice is composed of 39 independent CTUs [59].

### 3.2. Source Channel Encoding

The compressed video bit-stream comprises three partitions types A, B, and C of all the slices per frame. The bit-stream $x_{i}$ is then mapped to bit-string $x_{i}^{\prime }$ using a special bitmapping scheme, referred to as Over-complete Mapping (OCM), where we have i=1,2,3…n. Thereafter the mapped bit string $x_{i}^{\prime }$ is interleaved to conquer the burst of error into $\bar{x_{i}}$ using the bit interleaver (Π). The interleaved sequence $\bar{x_{i}}$ is then passed to the RSC encoder which encodes the sequence by different code rates as shown in Table 3. Our proposed NCC and NCDSTS-SP schemes employ Rate-1 OCM, presented in Table 2, as inner code while Rate-1/3 RSC code is employed as outer channel code. Contrary to this and in order to improve the convergence behavior of our employed iterative coding, CDSTS-SP scheme employ Rate-3/4 OCM, presented in Table 2, as inner code while Rate-4/9 RSC code is employed as outer channel code. The channel coded output from this block is forwarded to the next block for further processing.

### 3.3. Modulation and Transmission

The encoded bit-stream received from the source channel encoding block is then forwarded to modulation and transmission block. Using NCC scheme the received bit-stream is mapped to the stream of QPSK symbols. Afterwards, the Single Input Single Output (SISO) transmitter antenna is used to transmit the QPSK modulated Symbols over Rayleigh Fading channels.

While using NCDSTS-SP and CDSTS-SP schemes the received bit-stream is mapped to the SP symbols $S_{i}$, where i=0,1,2,3…L−1 by SP mapper, *L* is the modulated symbols that represents the number of SP signaling elements [52,60]. Afterwards, the DSTS transmitter antenna is used to transmit the SP modulated Symbols over Rayleigh Fading channels.

DSTS transceiver is liable to diversity gain in wireless network, reduced BER and accomplished a substantial video quality improvement. Due to embedding DSTS MIMO scheme, the proposed transceivers can be deployed in diverse environment for providing better video quality performance.

At the decoder side the received signal is first presented to the DSTS decoder and SP demapper which gives the Log-Likelihood Ratios (LLRs) value for the bit-stream. The generated LLR values are forwarded to the next block for further processing.

### 3.4. Iterative Soft Decoding

Subsequently, the extrinsic LLRs information received from the modulation and transmission block is then exchanged between the RSC decoders and SBSD in iterative fashion. The extrinsic information for Markov zero order model using SBSD can be found in [61]. The residual redundancy remains in M-ary symbols after the source encoding by H.264 and H.265 in term of non-uniform probability distribution $P[S_{n}\left(j\right)]$, where $S_{n}\left(j\right)=\left[S_{n}\left(1\right),S_{n}\left(2\right),S_{n}\left(3\right)\ldots S_{n}\left(M\right)\right]$ refer to Table 2. The results in Table 2 is accomplished by assigning 3 bits/symbols to the H.264/AVC encoded bit-stream, i.e., $\left[b_{1}\left(1\right),b_{1}\left(2\right),b_{1}\left(3\right);b_{2}\left(1\right),b_{2}\left(2\right),b_{2}\left(3\right)\ldots b_{j}\left(3\right)\right]$. The channel output symbol is generated by the following expression, provided that the bits are independent of each other.

(24) $$\begin{array}{c} \;P\left[{\hat{y}}_{\tau }|y_{\tau }\right]=\prod_{k=1}^{K}P\left[{\hat{y}}_{\tau }\left(k\right)|y_{\tau }\left(k\right)\right], \end{array}$$

where ${\hat{y}}_{\tau }$ is the received *K*-bit sequence on transmission of $y_{\tau }$. For a specific bit $\left[y_{\tau }\left(\lambda \right)\right]$, the output extrinsic channel information $P[{\hat{y}}_{\tau }^{\left[ext\right]}|y_{\tau }^{\left[ext\right]}]$ can be achieved as follow.

(25) $$P\left[{\hat{y}}_{\tau }^{\left[ext\right]}|y_{\tau }^{\left[ext\right]}\right]=\prod_{k=1,k\neq \lambda }^{K}P\left[{\hat{y}}_{\tau }\left(k\right)|y_{\tau }\left(k\right)\right].$$

Eventually, the channel output information is combined with the A-priori knowledge of that corresponding symbol to achieve the resultant LLR extrinsic value, as expressed below.

(26) $$\begin{array}{c} \;LLR[y_{\tau }\left(\lambda \right)]= \end{array}$$

$$\begin{array}{c} \;log\left(\frac{\sum_{y_{\tau }^{\left[ext\right]}}P\left[y_{\tau }^{\left[ext\right]}|y_{\tau }\left(\lambda \right)=+1\right].P\left[{\hat{y}}_{\tau }^{\left[ext\right]}|y_{\tau }^{\left[ext\right]}\right]}{\sum_{y_{\tau }^{\left[ext\right]}}P\left[y_{\tau }^{\left[ext\right]}|y_{\tau }\left(\lambda \right)=-1\right].P\left[{\hat{y}}_{\tau }^{\left[ext\right]}|y_{\tau }^{\left[ext\right]}\right]}\right). \end{array}$$

Natural redundancy residue in the encoded bit stream $x_{i}$ and is used in conventional SBSD as source of performance to obtain the extrinsic information. The achievable performance in video bit stream is limited when coding standard H.264/H.265 is used as encoder, which remove most of the residual redundancy. Therefore, the proposed scheme uses over complete mapping to generate artificial redundancy in coded video bit stream. To extract extrinsic information, the ingredient inner and outer decoder of iterative channel decoding provides sufficient information to each other in each iteration using the concept of interlacing.

EXIT chart operates on the special property of iterative decoder, which is convergence behavior as proposed by Stephen ten Brink [62], by exploiting the exchange of input and output mutual information. It is useful for serial, parallel and hybrid concatenated systems as well. Our proposed OCM is designed for employing this specific property of EXIT charts. If there is an open tunnel between the EXIT curves of the RSC and SBSD decoder, then the iterative decoding achieve extremely small decoded BER. Consequently, the above condition becomes satisfied when the EXIT curve intersects at a point $\left(I_{A},I_{B}\right)=\left(1,1\right)$ and it require a hamming distance between any two codewords $(d_{H},min)=2$, which is briefly explained in [26,27,28]. This condition stimulates our OCM scheme that it is possible to find a code table that satisfies the above condition by complete search of all codewords having mapping rate reciprocal to that of code rate and having $(d_{H},min)=2$, as presented in Table 4. Based on this table, the EXIT curve gets closer to the perfect point of convergence i.e., reaching to $\left(I_{A},I_{E}\right)=\left(1,1\right)$ of both the OCM and codec block, regardless of the shape of the Exit curve of the standalone encoder.

**Figure 5:**
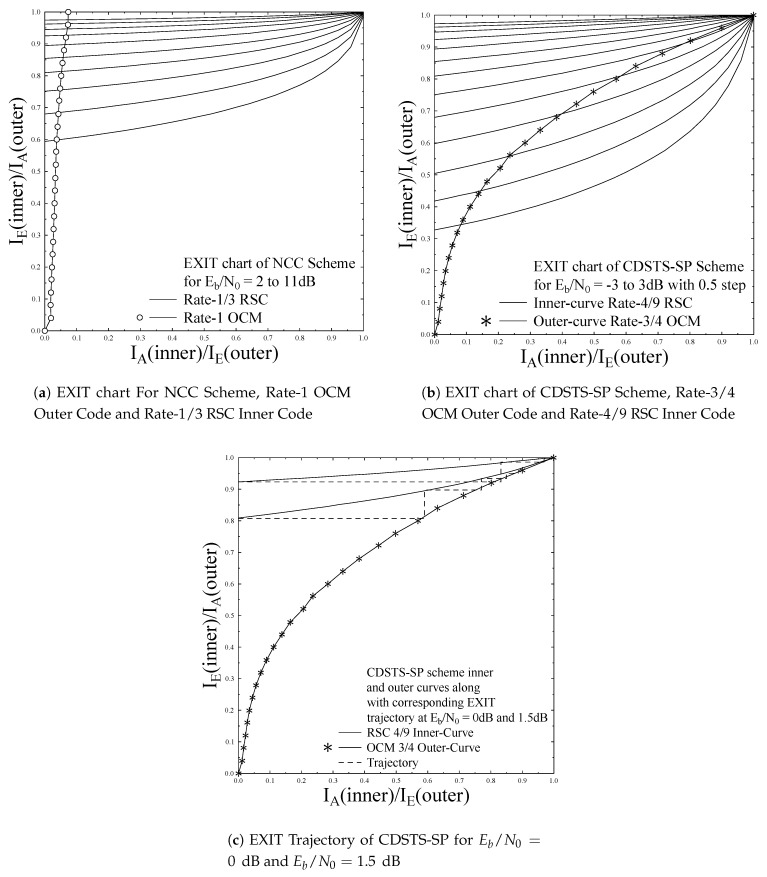
EXIT chart comparison for the proposed schemes.

Moreover, the EXIT curve for NCC scheme of SBSD using OCM rate-1 inner code and RSC rate-1/3 outer code is shown in Figure 5a. It can be observed that OCM rate-1 is not capable to reach to the point of perfect convergence—i.e., to the rightmost corner at ($I_{A}$; $I_{E}$) = (1, 1) and hence is not capable to exploit a beneficial advantage of iterative decoding. In contrast, the EXIT curve of SBSD using OCM rate-3/4 inner code reaches the point of perfect convergence, which in combination with Rate-4/9 RSC code can get advantage of iterative decoding by iteratively exchanging extrinsic information to achieve very low BER refer to Figure 5b. The EXIT Trajectory using OCM Rate-3/4 along with Rate-4/9 RSC code of CDSTS-SP at $E_{b}/N_{0}$ of −3 to 3 dB is shown in Figure 5c. It can be observed that due to convergence of inner and outer code to the point of perfect convergence—i.e., at $\left(I_{A};I_{E}\right)=\left(1,1\right)$, iterative decoding is capable to iteratively reach at the point of perfect convergence through an open-tunnel available at 0,1.5dB$E_{b}/N_{0}$ and above.

## 4. Simulations Results and Analysis

**Figure 6:**
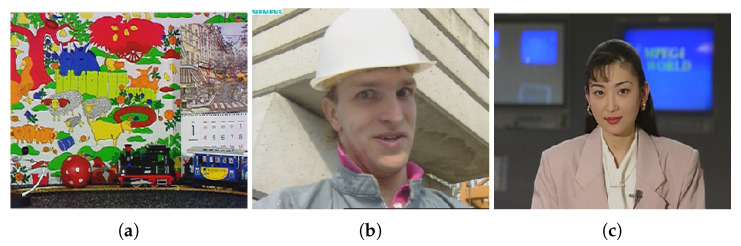
Video Sequences (**a**) MOBILE; (**b**) FOREMAN; (**c**) AKIYO.

This section provides a detailed performance analysis of the proposed system. The JM version 15 video and HM master codec developed by the joint video team (JVT) is used as a reference H.264 and H.265 source encoder. The proposed systems is simulated using IT++ signal processing and communications library, coded in C++. To perform a fair analysis between the three schemes, several aspects of the proposed systems have been considered. Various sample of the diverse video sequences is considered and provided as an input to the considered system models. Convergence behavior in iterative source channel decoding is specifically analyzed in the proposed work with different code rates of the inner and outer channel codes. In our simulation, three different video sequences—i.e., AKIYO, FOREMAN and MOBILE as shown in Figure 6, each with three different resolution (CIF, QCIF and 4CIF) in YUV(4:2:0) format is used as testing sequences.

Brief information about the video sequences used in the simulation experiments is as follows:

- Akiyo: This is a video of newscaster with extremely negligible movement and less details in the background.
- Foreman: This is a video of a foreman with rapidly moving face, extensive zoom out, average details and regular structures.
- Mobile: This is a video of a moving toy train with high details and dynamism.

The idea of using these different diverse video sequences with different video resolutions, level of motion and dynamism is to investigate the impact of video contents on its objective video quality while considering similar channel. As the extent of compression and robustness of the coded bit-stream is directly linked with its resolution, dynamism and motion content of the video stream, therefore the objective video quality performance of different video sequences is expected to be variable, while considering similar communication setup.

The number of bits per frame for luminance and chrominance in different sampling format and video sequences are calculated from [5] as depicted in Table 5. The duration of each video comprising of 45 frames at the frame rate of 15 fps is considered. The macro-blocks in H.264/AVC are processed in the raster scan order of a group of MBs called a slice, which represents a region of a given picture that can be processed independently of each other. In our experimental setup each slice consists of 11 Mbs while in HEVC, each frame is divided into slices which comprises CTUs. The considered slice types are intra (I), inter (P) slices. All MBs and CTUs in I slice are encoded using intra mode where as in P all MBs are coded using Intra coded mode with reference to previous frame. The encoder H.264.AVC is set at 15 fps. The resultant video frame sequence followed the pattern $I_{1}P_{2}P_{3}P_{4}\ldots P_{14}$ in which each 15th frame is I and the predecessor 14 are p frames. All the simulation parameters are listed in Table 1 and Table 3.

In order to reduce the computational complexity, iterations between RSC and SBSD decoders are set to 3 and 5 for the OCM rate-1 and $\frac{3}{4}$ respectively. The averaged results are obtained by repeating every 45 frames 160 times. The proposed system performance is exploited while considering the same overall code rate for the input H.264 encoded bit-stream.

The measurement of video quality is a major task in a scenario where there is a transmission of a compressed video over noisy channels. Subjective [63] and objective [63] techniques are widely used for measuring the video quality. Human participants are required in subjective technique to assess the streaming quality and therefore it is a time-consuming solution. On the other hand, PSNR is an objective metric for measuring the video quality when the video contents, codec and underlying communication setup remains unchanged [64].

PSNR is the ratio between the signal of the power of the original signal to that of the corrupted signal. PSNR is traditionally used as a quality metric for evaluating algorithms in multimedia streaming systems. It is generally expressed in term of Mean Square Error (MSE). The MSE of an image *X* having dimension of size M×N and its corrupted image *Y* can be expressed by the following equation.

(27) $$MSE=\frac{1}{M*N}\sum_{i=1}^{M}\sum_{j=1}^{N}\left[X\left(i,j\right)-Y\left(i,j\right)\right]$$

(28) $$PSNR\left(dB\right)=10\times \log_{10}\frac{MAX_{X}}{MSE}$$

where $MAX_{X}$ is the maximum value of a pixel of image *X* and can be calculated as $2^{B}-1$ for a *B* bits per sample.

The BER and PSNR performance of the simulation work were plotted against varying $E_{b}/N_{0}$ values, while utilizing diverse video sequences having different resolution, motion and dynamism while employing rayleigh fading channel as a communication medium.

In our simulation scenarios OCM is employed as inner code, while RSC is used as outer channel code. Furthermore, in the considered NCC scheme QPSK modulation is employed, while in NCDSTS-SP and CDSTS-SP sphere packing modulation is used in combination with DSTS.

**Figure 7:**
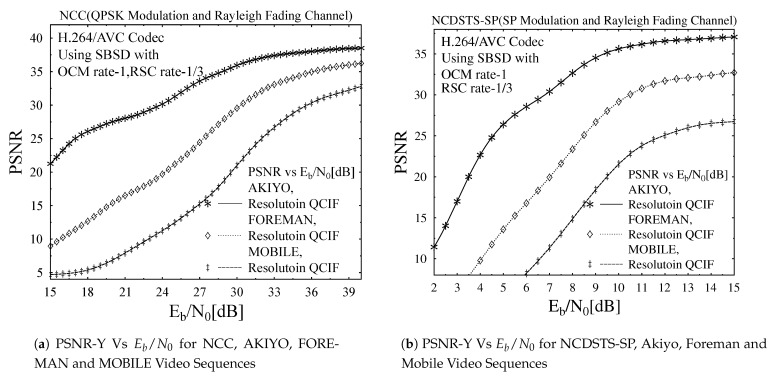

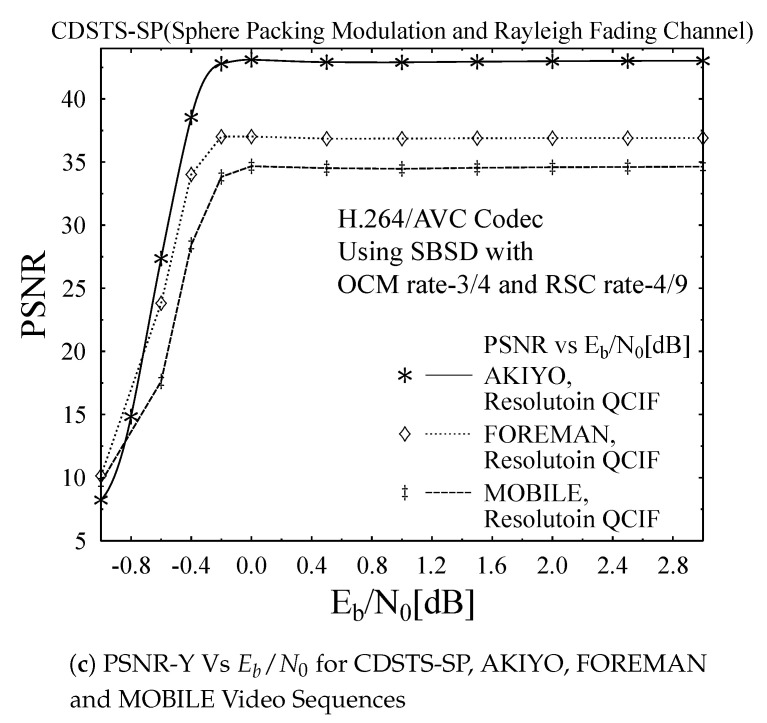
PSNR and BER comparison for the proposed schemes.

Furthermore, in order to analyze the impact of varying motion contents and dynamism of video scene on the objective video quality performance of the transmission scheme, its PSNR versus $E_{b}/N_{0}$ dB performance trends are plotted in Figure 7a–c for NCC, NCDSTS-SP and CDSTS-SP schemes, respectively, while using AKIYO, FOREMAN and MOBILE video sequences in its QCIF video resolution. In Figure 7a–c similar trend in performance variations due to employment of different video sequences, with varying motion contents and dynamism is observed for all three employed schemes. Frome Figure 7a, the PSNR Vs $E_{B}/N_{0}$ [dB] is plotted for the same resolution resolution of different sequences i.e., AKIYO, FOREMAN and MOBILE considering NCC scheme. More specifically, it is observed from Figure 7a–c that the presence of high channel noise, the AKIYO video sequence with low motion contents and dynamism outperforms the FOREMAN and MOBILE video sequence with high motion contents and dynamism. This is due to the fact that in the presence of low motion contents and dynamism of video, the H.264/AVC can compress the corresponding video sequence more robustly within the allocated bit-rate budget. Furthermore, the performance of the H.264/AVC error concealment mechanism is more effective in video sequences with low motion contents and dynamism and as a result its objective quality performance will outperform.

**Figure 8:**
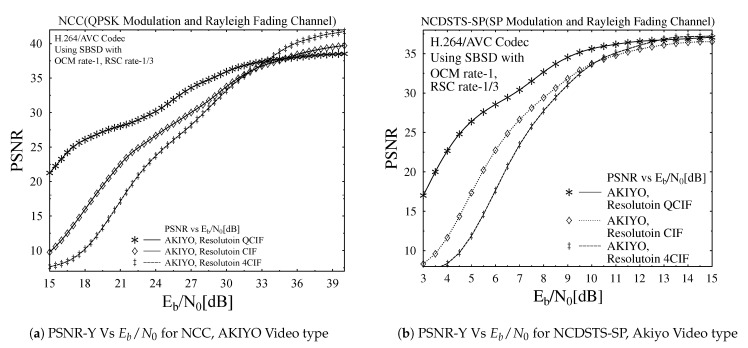

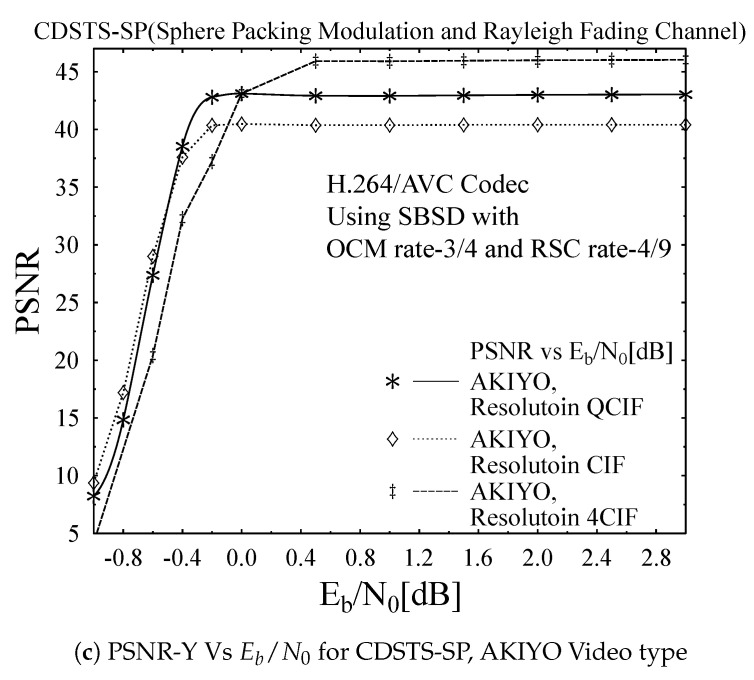
PSNR comparison for the proposed schemes.

In order to analyze the impact of varying video resolution on the objective video quality performance of the transmission scheme its PSNR versus $E_{b}/N_{0}$ dB performance trends were plotted in Figure 8a–c for NCC, NCDSTS-SP and CDSTS-SP schemes, respectively, while using AKIYO video sequence. Figure 8a is plotted for transmitting AKIYO video sequence on NCC scheme from only one Tx antenna varying the $E_{b}/N_{0}$ values. As it is clear that the for a higher $E_{b}/N_{0}$, the performance of the NCC scheme in terms of PSNR is reasonable for all the three video resolutions. Moreover, due to using fast fading channel, lower the resolution of the video sequence higher its performance while NCC does not employing any differential detection at the Rx antenna. Most of the time, the SNR comes under deep fade for which the decoder cannot reliably decoder the received signal and hence the performance is degraded for the concerned scheme. In contrast, the performances of NCDSTS-SP and CDSTS-SP schemes for a very low $E_{b}/N_{0}$ values are much better due to using differential detection and spreading codes as shown in Figure 8b,c. Moreover, CDSTS-SP scheme outperform in terms of PSNR values compare to NDSTS-SP due to the reason that CDSTS-SP achieved perfect convergence on EXIT chart using the specific inner and outer code rates specified at both the encoders. Therefore, the rule of code rate is visible on quality performance upon using the iterative decoding. The performance difference in terms of resolution used is still valid for which both the schemes outperform for lower resolution. In Figure 8a–c similar trend in performance variations due employment of different resolution videos is observed for all three employed schemes. As from Figure 8c, the PSNR reached to its peak value for a lower $E_{b}/N_{0}$ value and remains constant for a remaining range of $E_{b}/N_{0}$. It is due to the fact that no matter how much the $E_{b}/N_{0}$ is increased, the performance remains constant after the $E_{b}/N_{0}$ range at which perfect convergence is achieved. Therefore, it is observed from Figure 8a–c that the presence of high channel noise the low resolution video outperforms its high resolution counterparts.

**Figure 9:**
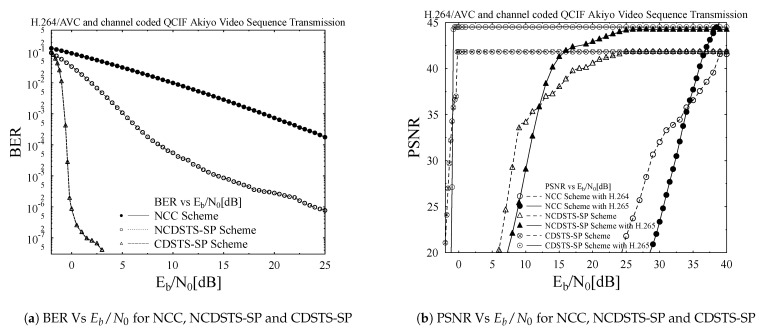
BER and PSNR comparison for the proposed schemes.

The BER versus $E_{b}/N_{0}$ dB performance of the NCC, NCDSTS-SP and CDSTS-SP schemes is presented in Figure 9a. BER performance is plotted against $E_{b}/N_{0}$ values for all the three schemes. The BER values approaches to $10^{-7}$ for a very low $E_{b}/N_{0}$ for CDSTS-SP and the same fact is visualize for PSNR from Figure 9b. As it is already discussed that CDSTS-SP is employing differential methods for recovering the SNR values at the receiver and is suitable scheme for fast fading channel while NCDST-SP is also employing differential methods but cannot obtained the perfect convergence at the considered inner and outer code rate. The worst BER performance is noticed for NCC scheme which is neither employing differential scheme nor obtained convergence on the specific inner and outer code rate. Its observed from Figure 9a that the BER performance of CDSTS-SP is best as compared to NCDSTS-SP scheme which is performing better then NCC scheme.

The effects of BER on PSNR performance of the CDSTS-SP scheme can be easily observed from Figure 9b. For a very low range of $E_{b}/N_{0}$ [dB] values, the PSNR reached to its peak values and remains constant for the rest of the $E_{b}/N_{0}$ range which shows that the differential scheme obtained a sufficient information about a channel on iterative decoding from observing the phases of the current and the the previous symbols that can estimate the value on a lower range of $E_{b}/N_{0}$ values. Similar performance is observed for NDSTS-SP which on slightly higher values of $E_{b}/N_{0}$ obtained convergence and tends to be constant for the rest of the $E_{b}/N_{0}$ range while the NCC scheme is worst compared to the others and showing an increasing trend when $E_{b}/N_{0}$ values increases. Finally, its observed from the PSNR versus $E_{b}/N_{0}$ dB performance curves of the NCC, NCDSTS-SP and CDSTS-SP schemes, presented in Figure 9b that the sophisticated system design of CDSTS-SP outperform its counterpart NCC, NCDSTS-SP schemes in terms of PSNR. More specifically, it is observed from Figure 9b that NCDSTS-SP results in PSNR gain of 6 dB and CDSTS-SP results in PSNR gain of 28 dB for $E_{b}/N_{0}$ value of 10 dB, with reference to bench marker system design of NCC.

The PSNR Vs *Eb*/*N*0 performance of the three advocated schemes—i.e., NCC, NCDSTS-SP and CDSTS-SP with reference to the employed H.264 and H.265 standards is presented in Figure 9b. It can be observed from the Figure 9b, that while utilizing H.265 video coding standard, the NCDSTS-SP with enhanced transmission mechanism results in $E_{b}/N_{0}$ gain of 20 dB with reference to the NCC coding and transmission mechanism at the objective PSNR value of 42 dB. It is important to mention that both NCC and NCDSTS-SP schemes have employed identical code rate. Furthermore, the CDSTS-SP with convergent coding mechanism achieved superior performance with reference to the competing NCDSTS-SP counterpart mechanism, with a performance gain of 16 dB at the objective PSNR grade of 42 dB. Moreover, it is observed that while employing any of the three advocated schemes—i.e., NCC, NCDSTS-SP and CDSTS-SP the maximum achievable PSNR gain through H.265 video coding standard is 45 dB, with a PSNR gain of 3 dB with reference to the identical code rate H.264 coding scheme.

## 5. Conclusions

This paper presents performance analysis of sophisticated channel coding and transmission schemes for reliable transmission of H.264/AVC and H.265/HEVC compressed video. Three different coding and transmission schemes were presented, namely Non-Convergent Coding (NCC), Non-Convergent Coding assisted with Differential Space Time Spreading (DSTS) and Sphere Packing (SP) modulation (NCDSTS-SP) and Convergent Coding assisted with Differential Space Time Spreading (DSTS) and Sphere Packing (SP) modulation (CDSTS-SP). Different diverse combination of source mapping techniques and channel coding techniques were employed and their convergence behavior is analyzed with the aid of Extrinsic Information Transfer (EXIT) Charts. Furthermore, to improve the diversity gain of the transceiver advanced Sphere Packing (SP) modulation technique assisted by Differential Space Time Spreading (DSTS) is employed. The BER and PSNR performance of the proposed schemes were plotted against varying $E_{b}/N_{0}$ values, while utilizing diverse video sequences having different resolution, motion and dynamism while employing Rayleigh fading channel as a communication medium. It was observed that in the presence of high channel noise the low resolution videos outperforms its high resolution counterparts. Furthermore, it was observed that in the presence of high channel noise, the AKIYO video sequence with low motion contents and dynamism outperforms the FOREMAN and MOBILE video sequence with high motion contents and dynamism. More specifically, it is observed that while utilizing H.265 video coding standard, the NCDSTS-SP scheme with enhanced transmission mechanism results in *Eb*/*N*0 gain of 20 dB with reference to the NCC coding and transmission mechanism at the objective PSNR value of 42 dB. It is important to mention that both NCC and NCDSTS-SP schemes have employed identical code rate. Furthermore, the CDSTS-SP with convergent coding mechanism achieved superior performance with reference to the equivalent rate NCDSTS-SP counterpart mechanism, with a performance gain of 16 dB at the objective PSNR grade of 42 dB. Moreover, it is observed that the maximum achievable PSNR gain through H.265 video coding standard is 45 dB, with a PSNR gain of 3 dB with reference to the identical code rate H.264 coding scheme. Furthermore, it is observed from that the BER and PSNR versus $E_{b}/N_{0}$ dB performance of the NCC, NCDSTS-SP and CDSTS-SP schemes that the BER performance of CDSTS-SP is best as compared to NCDSTS-SP scheme, which is performing better then NCC scheme. More specifically, it is observed that the NCDSTS-SP results in PSNR gain of 20 dB with reference to the NCC coding and transmission mechanism at the objective PSNR value of 42 dB. Furthermore, the CDSTS-SP with convergent coding mechanism achieved superior performance with reference to the equivalent rate NCDSTS-SP counterpart mechanism, with a performance gain of 16 dB at the objective PSNR grade of 42 dB. Moreover, it is observed that the maximum achievable PSNR gain through H.265 video coding standard is 45 dB, with a PSNR gain of 3 dB with reference to the identical code rate H.264 coding scheme.

## Figures and Tables

**Table 1:** Parameters settings of the source encoder.

Video Coding	Parameters
Compression standard	H.264 & H.265
Date-Rate for both H.264 and H.265	64 kbps
Frame-Rate	15 fps
Number of Slices Per frame in H.264	9
Macro-Blocks Per Slice in H.264	11
Macro-Blocks per frame in Intra-frame in H.264	3
Profile in H.264	Extended
IntraPeriod of I-pictures	15
PartitionMode in H.264	3 Partitions per Slice
Entropy coding method in H.264	UVLC
SliceMode in H.264	Fixed # of MBs in slice
SOA	1
Slice Mode	1
Number of CTUs in slice 1 and slice 2	99
MaxCUWidth	64
MaxCUHeight	64
Quantization Parameter	32

**Table 2:** Probabilities of Symbols with OCM Rate-1 and Rate-$\frac{3}{4}$.

Symbols	OCM Rate-1	OCM Rate-$\frac{3}{4}$
000	000	0000
001	001	1001
010	010	1010
011	011	0011
100	100	1100
101	101	0101
110	110	0110
111	111	1111

**Table 3:** Parameters settings of the transceivers.

System	Parameters		
	NCC	NCDSTS-SP	CDSTS-SP
Ourter Code	Rate-1 OCM	Rate-1 OCM	Rate-3/4 OCM
Inner Code	Rate-1/3 RSC	Rate-1/3 RSC	Rate-4/9 RSC
Modulation	QPSK	SP	SP
MIMO Scheme	Nill	DSTS	DSTS
Tx	1	2	2
Rx	1	1	1
Channel	Rayleigh	Rayleigh	Rayleigh
Doppler Frequency	0.01	0.01	0.01

**Table 4:** Code rate and modulation technique in the prposed models.

Scheme	Bit Rate		Modulation
	Outer Code	Inner Code	
NCC	Rate-1 OCM	Rate-1/3 RSC code	QPSK
NCDSTS-SP	Rate-1 OCM	Rate-1/3 RSC code	Sphere Packing
CDSTS-SP	Rate-3/4 OCM	Rate-4/9 RSC code	Sphere Packing

**Table 5:** Analysis of different sampling formats and video sequences.

Sampling Format	Video Sequence Resolution	Luminance (Y) Resolution	Luminance Bits per Frame	Chrominance (Cb & Cr) Resolution	Chrominance Bits per Frame
YUV(4:4:4)	QCIF	176 × 144	608,256	176 × 144	608,256
	CIF	352 × 288	2,433,024	352 × 288	2,433,024
	4CIF	704 × 576	9,732,096	704 × 576	9,732,096
YUV(4:2:2)	QCIF	176 × 144	405,504	88 × 144	202,752
	CIF	352 × 288	1,422,016	176 × 288	811,008
	4CIF	704 × 576	6,488,064	352 × 576	3,244,032
YUV(4:2:0)	QCIF	176 × 144	304,128	88 × 72	76,032
	CIF	352 × 288	1,216,512	176 × 144	304,128
	4CIF	704 × 576	4,866,044	352 × 288	1,216,512
Video Sequence (VS)	Frames	Frame rate	Reason for a selection		
AIYO	45	15 fps	Low motion and dynamism		
FOREMAN			Medium motion and dynamism		
MOBILE			High motion and dynamism		

## Data Availability

Not applicable.

## References

[1] Index C. (2016). Cisco visual networking index: Global mobile data traffic forecast update, 2015–2020. Cisco Technical Report.

[2] Global Mobile Data Traffic Forecast (2020). Cisco Visual Networking Index: Global Mobile Data Traffic Forecast Update, 2018–2023.

[3] Björnson E., Hoydis J., Sanguinetti L. (2017). Massive MIMO networks: Spectral, energy, and hardware efficiency. Found. Trends Signal Process..

[4] Pirinen P. A brief overview of 5G research activities. Proceedings of the 1st International Conference on 5G for Ubiquitous Connectivity.

[5] Richardson I.E. (2004). H. 264 and MPEG-4 Video Compression: Video Coding for Next-Generation Multimedia.

[6] Shannon C.E. (1948). A Mathematical Theory of Communication. Bell Syst. Tech. J..

[7] Shannon C.E. (1949). Communication in the Presence of Noise. Proc. IRE.

[8] Hamming R.W. (1950). Error Detecting and Error Correcting Codes. Bell Syst. Tech. J..

[9] Elias P. (1955). Coding for noisy channels. IRE Convention Record.

[10] Nguyen H.V., Xu C., Ng S.X., Hanzo L. (2015). Near-capacity wireless system design principles. IEEE Commun. Surv. Tutor..

[11] Hagelbarger D.W. (1959). Recurrent Codes: Easily Mechanized, Burst-Correcting, Binary Codes. Bell Syst. Tech. J..

[12] Wozencraft J.M., Reiffen B. (1961). Sequential Decoding.

[13] Fano R. (1963). A heuristic discussion of probabilistic decoding. IEEE Trans. Inf. Theory.

[14] Viterbi A. (1967). Error bounds for convolutional codes and an asymptotically optimum decoding algorithm. IEEE Trans. Inf. Theory.

[15] Forney G.D. (1973). The viterbi algorithm. Proc. IEEE.

[16] Forney G.D. (2005). The viterbi algorithm: A personal history. arXiv.

[17] Forney G. (1972). Maximum-likelihood sequence estimation of digital sequences in the presence of intersymbol interference. IEEE Trans. Inf. Theory.

[18] Bahl L., Cullum C., Frazer W., Jelinek F. (1972). An efficient algorithm for computing free distance (Corresp.). IEEE Trans. Inf. Theory.

[19] Bahl L., Cocke J., Jelinek F., Raviv J. (1974). Optimal decoding of linear codes for minimizing symbol error rate (Corresp.). IEEE Trans. Inf. Theory.

[20] Berrou C., Glavieux A., Thitimajshima P. Near Shannon limit error-correcting coding and decoding: Turbo-codes. 1. Proceedings of the ICC 93—IEEE International Conference on Communications.

[21] Berrou C., Glavieux A. (2007). Near optimum error correcting coding and decoding: Turbo-codes. The Best of the Best: Fifty Years of Communications and Networking Research.

[22] Steele R., Hanzo L. (1999). Mobile Radio Communications: Second and Third Generation Cellular and WATM Systems.

[23] Koch W., Baier A. Optimum and sub-optimum detection of coded data disturbed by time-varying intersymbol interference (applicable to digital mobile radio receivers). Proceedings of the GLOBECOM 90: IEEE Global Telecommunications Conference and Exhibition.

[24] Chen C., Wang L., Lau F.C. (2018). Joint optimization of protograph LDPC code pair for joint source and channel coding. IEEE Trans. Commun..

[25] Burth Kurka D., Gündüz D. (2020). Joint Source-Channel Coding of Images with (not very) Deep Learning. International Zurich Seminar on Information and Communication (IZS 2020) Proceedings.

[26] Minallah N., Ullah K., Frnda J., Cengiz K., Awais Javed M. (2021). Transmitter Diversity Gain Technique Aided Irregular Channel Coding for Mobile Video Transmission. Entropy.

[27] Minallah N., Butt M.F.U., Khan I.U., Ahmed I., Khattak K.S., Qiao G., Liu S. (2020). Analysis of Near-Capacity Iterative Decoding Schemes for Wireless Communication Using EXIT Charts. IEEE Access.

[28] Minallah N., Ahmed I., Ijaz M., Khan A.S., Hasan L., Rehman A. (2021). On the Performance of Self-Concatenated Coding for Wireless Mobile Video Transmission Using DSTS-SP-Assisted Smart Antenna System. Wirel. Commun. Mob. Comput..

[29] Brejza M.F., Maunder R.G., Al-Hashimi B.M., Hanzo L. (2017). A High-Throughput FPGA Architecture for Joint Source and Channel Decoding. IEEE Access.

[30] Balsa J., Domínguez-Bolaño T., Fresnedo Ó., García-Naya J.A., Castedo L. (2019). Transmission of Still Images Using Low-Complexity Analog Joint Source-Channel Coding. Sensors.

[31] Bourtsoulatze E., Burth Kurka D., Gündüz D. (2019). Deep Joint Source-Channel Coding for Wireless Image Transmission. IEEE Trans. Cogn. Commun. Netw..

[32] Yang H., Qing L., He X., Ou X., Liu X. (2018). Robust distributed video coding for wireless multimedia sensor networks. Multimed. Tools Appl..

[33] Cai S., Lin W., Yao X., Wei B., Ma X. (2020). Systematic Convolutional Low Density Generator Matrix Code. arXiv.

[34] Hagenauer J. The exit chart—Introduction to extrinsic information transfer in iterative processing. Proceedings of the 2004 12th European Signal Processing Conference.

[35] Brink S. (2000). Designing iterative decoding schemes with the extrinsic information chart. AEU Int. J. Electron. Commun.

[36] Du J., Yang L., Yuan J., Zhou L., He X. (2017). Bit Mapping Design for LDPC Coded BICM Schemes with Multi-Edge Type EXIT Chart. IEEE Commun. Lett..

[37] Stockhammer T., Hannuksela M., Wiegand T. (2003). H. 264/AVC in wireless environments. IEEE Trans. Circuits Syst. Video Technol..

[38] Nam C., Chu C., Kim T., Han S. (2020). A novel motion recovery using temporal and spatial correlation for a fast temporal error concealment over H. 264 video sequences. Multimed. Tools Appl..

[39] Nie H., Jiang X., Tang W., Zhang S., Dou W. (2020). Data security over wireless transmission for enterprise multimedia security with fountain codes. Multimed. Tools Appl..

[40] Yuan J. (2019). Video data wireless transmission method based on cross-layer bitrate adaptation and error control. Multimed. Tools Appl..

[41] Ibrahim S.K., Khamiss N.N. (2019). A new wireless generation technology for video streaming. J. Comput. Netw. Commun..

[42] Ma Z., Sun S. (2020). Research on HEVC screen content coding and video transmission technology based on machine learning. Ad Hoc Netw..

[43] Choi Y., Joo J. (2014). Exploration of practical HEVC/H. 265 sample adaptive offset encoding policies. IEEE Signal Process. Lett..

[44] Ichigaya A., Nishida Y. (2016). Required bit rates analysis for a new broadcasting service using HEVC/H. 265. IEEE Trans. Broadcast..

[45] Chen Y., Chen K., Yuan S., Kuo S. (2016). Moving Object Counting Using a Tripwire in H.265/HEVC Bitstreams for Video Surveillance. IEEE Access.

[46] Hsieh J., Cai J., Wang Y., Guo Z. (2019). ML-Assisted DVFS-Aware HEVC Motion Estimation Design Scheme for Mobile APSoC. IEEE Syst. J..

[47] Singhadia A., Mamillapalli M., Chakrabarti I. (2020). Hardware-efficient 2D-DCT/IDCT architecture for portable HEVC-compliant devices. IEEE Trans. Consum. Electron..

[48] Coding H.E.V., Series H. (2013). Audiovisual and Multimedia Systems. Infrastructure of Audiovisual Services Coding Moving Video.

[49] Jafarkhani H. (2005). Space-Time Coding: Theory and Practice.

[50] Kułakowski P. (2006). The Multiple-Input Multiple-Output Systems in Slow and Fast Varying Radio Channels. Ph.D. Thesis.

[51] Tarokh V., Seshadri N., Calderbank A.R. (1998). Space-time codes for high data rate wireless communication: Performance criterion and code construction. IEEE Trans. Inf. Theory.

[52] Su W., Safar Z., Liu K.R. (2003). Space-time signal design for time-correlated Rayleigh fading channels. IEEE Int. Conf. Commun..

[53] Su W., Xia X.G. (2003). On space-time block codes from complex orthogonal designs. Wirel. Pers. Commun..

[54] El-Hajjar M.H. (2008). Near-Capacity MIMOs Using Iterative Detection. Ph.D. Thesis.

[55] Ohm J.R., Sullivan G.J., Schwarz H., Tan T.K., Wiegand T. (2012). Comparison of the coding efficiency of video coding standards—Including high efficiency video coding (HEVC). IEEE Trans. Circuits Syst. Video Technol..

[56] Sullivan G.J., Boyce J.M., Chen Y., Ohm J.R., Segall C.A., Vetro A. (2013). Standardized extensions of high efficiency video coding (HEVC). IEEE J. Sel. Top. Signal Process..

[57] Sullivan G.J., Ohm J.R., Han W.J., Wiegand T. (2012). Overview of the High Efficiency Video Coding (HEVC) Standard. IEEE Trans. Circuits Syst. Video Technol..

[58] Pourazad M.T., Doutre C., Azimi M., Nasiopoulos P. (2012). HEVC: The new gold standard for video compression: How does HEVC compare with H. 264/AVC?. IEEE Consum. Electron. Mag..

[59] Telecommunication Standardization Sector (2013). ITU-T Recommendation H. 265: High Efficiency Video Coding.

[60] El-Hajjar M., Alamri O., Ng S.X., Hanzo L. (2008). Turbo Detection of Precoded Sphere Packing Modulation Using Four Transmit Antennas for Differential Space-Time Spreading. IEEE Trans. Wirel. Commun..

[61] Adrat M., Vary P. (2005). Iterative source-channel decoding: Improved system design using EXIT charts. EURASIP J. Adv. Signal Process..

[62] Ten Brink S. (2001). Convergence behavior of iteratively decoded parallel concatenated codes. IEEE Trans. Commun..

[63] RECOMMENDATION ITU-R BT (2002). Methodology for the Subjective Assessment of the Quality of Television Pictures.

[64] Huynh-Thu Q., Ghanbari M. (2008). Scope of validity of PSNR in image/video quality assessment. Electron. Lett..

